# Uterine Angiomyolipoma Presenting as a Rapidly Growing Uterine Mass in a Postmenopausal Woman

**DOI:** 10.3390/diagnostics15232995

**Published:** 2025-11-25

**Authors:** Dae Hyun Song, Hyo Jung An, Jong Chul Baek

**Affiliations:** 1 Department of Pathology, Institute of Health Science, School of Medicine, Gyeongsang National University, Jinju 52727, Republic of Korea; golgy@hanmail.net (D.H.S.); ariel2020@naver.com (H.J.A.); 2 Department of Pathology, Gyeongsang National University Changwon Hospital, Changwon 51472, Republic of Korea; 3 Department of Obstetrics and Gynecology, Institute of Health Science, School of Medicine, Gyeongsang National University, Jinju 52727, Republic of Korea; 4 Department of Obstetrics and Gynecology, Gyeongsang National University Changwon Hospital, Changwon 51472, Republic of Korea

**Keywords:** uterine angiomyolipoma, perivascular epithelioid cell tumor (PEComa), nuclear atypia, smooth muscle tumor, postmenopausal uterine mass

## Abstract

Uterine angiomyolipoma (AML) is an exceptionally rare mesenchymal tumor of the perivascular epithelioid cell tumor (PEComa) family. Most cases are benign and exhibit a triphasic histologic pattern. Although extragenital PEComas typically show strong, diffuse HMB-45 reactivity, uterine AMLs/PEComas often exhibit weak or negative staining, thereby introducing diagnostic uncertainty. We describe a rare case of uterine AML with diffuse nuclear atypia in a postmenopausal woman, which mimicked a degenerative leiomyoma or leiomyosarcoma. A 49-year-old postmenopausal woman presented with the rapid enlargement of a uterine mass that had been followed for four years as a presumed leiomyoma. Imaging revealed a well-circumscribed uterine mass with heterogeneous enhancement, cystic degeneration, and restricted diffusion on MRI. A total hysterectomy was performed. Grossly, the tumor measured 8 cm. Microscopically, it consisted of pleomorphic epithelioid cells (70%), mature adipose tissue (20%), and thick-walled vessels. Immunohistochemistry revealed diffuse smooth muscle actin (SMA) positivity, while Human Melanoma Black (HMB)-45 and Melan-A were negative. Only one mitosis per 50 HPF was identified, with no atypical mitoses or necrosis, and the Ki-67 index was low (<5%). The patient has remained disease-free for 56 months post-surgery. This case represents the first documented HMB-45-negative uterine angiomyolipoma with diffuse nuclear atypia, characterized by a low mitotic index, low Ki-67 proliferation rate, and a benign 56-month follow-up. It broadens the morphologic spectrum of uterine AML, demonstrating that diffuse nuclear atypia can occur in HMB-45-negative tumors with benign behavior, and that atypia alone should not be interpreted as evidence of malignancy. Recognition of this rare variant is essential to avoid misdiagnosing it as leiomyosarcoma.

**Figure 1:**
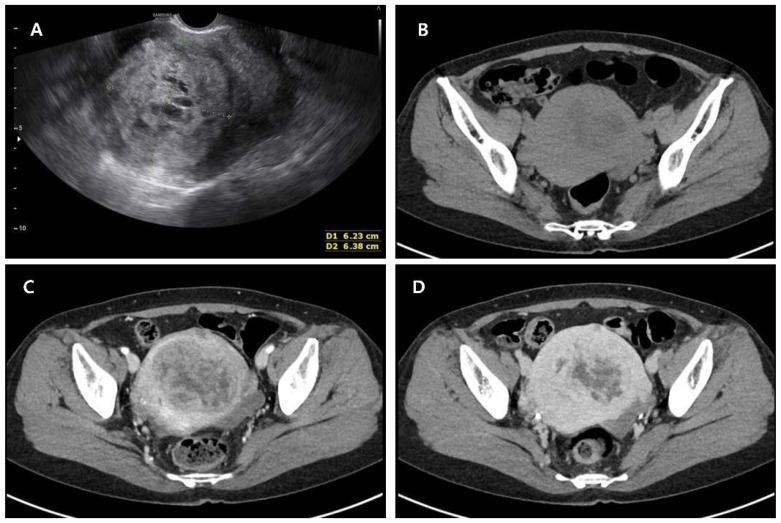
USG and Computed Tomography images of uterine mass. A 49-year-old postmenopausal woman (gravida 3, para 0) was referred to our institution for evaluation of a rapidly enlarging uterine mass that had been followed for four years as a presumed leiomyoma. She had experienced menopause three years prior and reported no abnormal vaginal bleeding or systemic symptoms. She had no significant underlying medical conditions and was not receiving any form of hormone replacement therapy. Transvaginal ultrasonography revealed an enlarged, heterogeneous echogenicity with irregular internal architecture, including focal cystic changes within the uterus, measuring approximately 6.23 × 6.38 cm in maximal diameter (**A**). On axial pre-enhanced CT image, a well-defined uterine mass with heterogeneous iso- to hypodense attenuation was seen, showing central low-density areas (**B**). On contrast-enhanced axial CT, the lesion showed heterogeneous enhancement, characterized by alternating solid enhancing areas and non-enhancing hypodense regions (**C**). On a delayed contrast-enhanced axial CT, the mass demonstrated a persistent enhancement of the solid areas of the tumor, with non-enhancing central portions corresponding to degeneration or necrosis (**D**). Ultrasonography revealed a large, well-circumscribed, heterogeneously hypoechoic uterine mass with cystic degeneration. CT imaging demonstrated a solid lesion with heterogeneous enhancement and central hypodense areas, and findings were compatible with a degenerative leiomyoma. However, these imaging features are not specific and may also be seen in uterine sarcomas, limiting the diagnostic value of ultrasound and CT in differentiating benign from malignant smooth muscle tumors.

**Figure 2:**
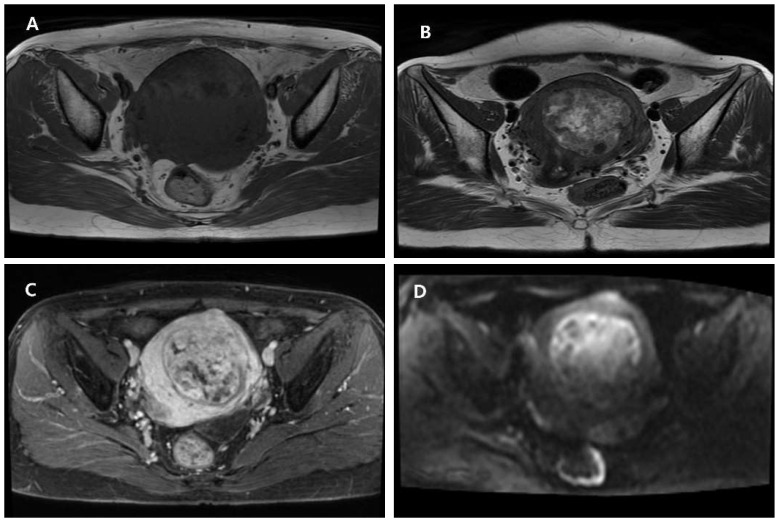
Magnetic resonance imaging (MRI) of the uterine mass. Axial T1-weighted image shows a well-defined lesion isointense to the myometrium without evidence of fat or hemorrhage (**A**). Axial T2-weighted image demonstrates a smooth, circumscribed margin and heterogeneous high T2 signal suggesting cystic or myxoid degeneration (**B**). Post-contrast T1-weighted image reveals heterogeneous enhancement with interspersed non-enhancing cystic areas (**C**). Diffusion-weighted image (DWI) shows a focal high signal within the solid component, indicating partial diffusion restriction (**D**). MRI demonstrated a well-circumscribed uterine mass with isointense T1 signal and heterogeneous high T2 signal compatible with cystic or myxoid degeneration. Post-contrast sequences showed heterogeneous enhancement with small non-enhancing components, and diffusion-weighted imaging revealed a focal area of partial diffusion restriction with intermediate ADC values. Although lipoleiomyomas typically contain overt macroscopic fat and PEComas often exhibit pronounced T2 hyperintensity with brisk early enhancement, the absence of these defining features—together with the coexistence of degenerative changes and focal diffusion restriction—ultimately yielded an indeterminate imaging impression. Taken together, these characteristics created a discordant radiologic profile: the smooth margins and T2 hyperintensity supported a benign leiomyoma with secondary degeneration, whereas the localized diffusion-restricted focus raised consideration of increased cellularity that may be encountered in more aggressive smooth muscle tumors. Such mixed and partly conflicting findings underscore the limitations of MRI in reliably distinguishing benign from malignant uterine smooth muscle neoplasms and highlight the need for histopathologic confirmation when imaging features remain equivocal. A total laparoscopic hysterectomy with bilateral salpingo-oophorectomy (BSO) was performed following thorough preoperative counseling. Because malignancy could not be excluded, power morcellation was avoided. The intact uterus was placed in an endoscopic retrieval bag and removed vaginally via colpotomy to prevent tumor spillage. No abnormal findings were observed in the pelvic cavity other than the enlarged uterus. A hysterectomy specimen was received for frozen section slide testing. During serial sectioning, a partially pinkish ivory-colored tumor measuring 6.7 × 8.2 cm was visually observed within the uterine myometrium. A portion of the tumor showed a soft, gelatinous consistency. Additionally, several small, typical, firm, white uterine leiomyomas were noted within the myometrium.

**Figure 3:**
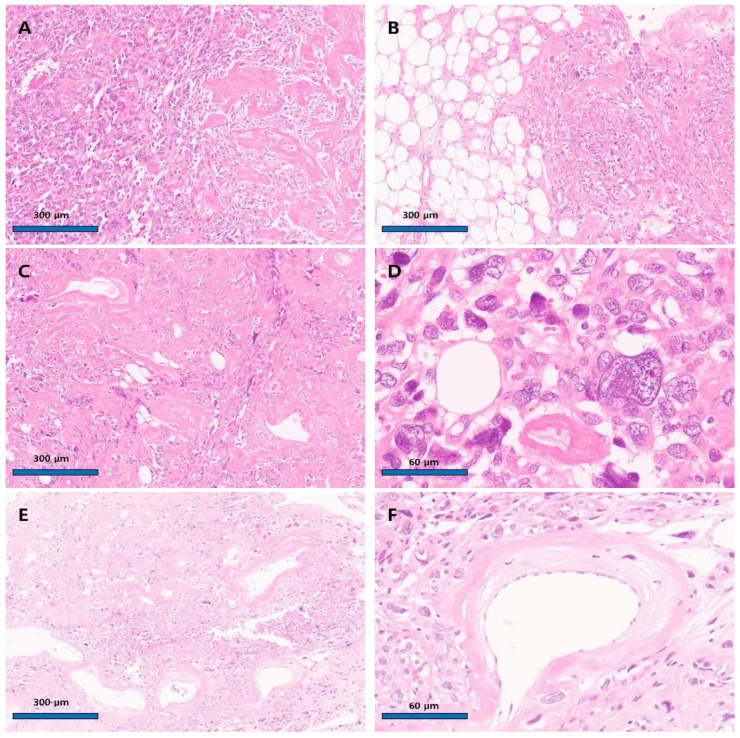
Microscopic finding of the uterine angiomyolipoma with nuclear atypism. (**A**) The tumor cells exhibited high cellular density and did not form any specific structures (**left panel**). The tumor cells showed an epithelioid morphology, and nuclear pleomorphism was observed. Acellular, hyalinized, tortuous structures were frequently seen (**right panel**) (×100, hematoxylin and eosin stain). (**B**) Areas of adipose cells were observed within the mass (**left panel**). (**C**) The mass contained tortuous vessels intermingled with scattered fat cells and tumor cells (×100, hematoxylin and eosin stain). (**D**) Large tumor cells were similar in size to fat cells (×400, hematoxylin and eosin stain). (**E**) In particular, the acellular hyalinized, convoluted structures were judged to be blood vessels and were frequently observed, leading to their identification as a tumor component (×100, hematoxylin and eosin stain). (**F**) A vessel lumen well-lined by endothelial cells (×400, hematoxylin and eosin stain). A representative section slide was prepared from the mass, showing pleomorphic epithelioid cells. No mitoses or tumor necrosis were observed. The frozen section slide test reported a possibility of smooth muscle tumors of uncertain malignant potential (STUMP). The remaining tumor specimen was fixed in formalin. Permanent sections revealed a triphasic tumor composed of pleomorphic epithelioid cells (70%), mature adipose cells (20%), and thick-walled, tortuous blood vessels (Figure 3).

**Figure 4:**
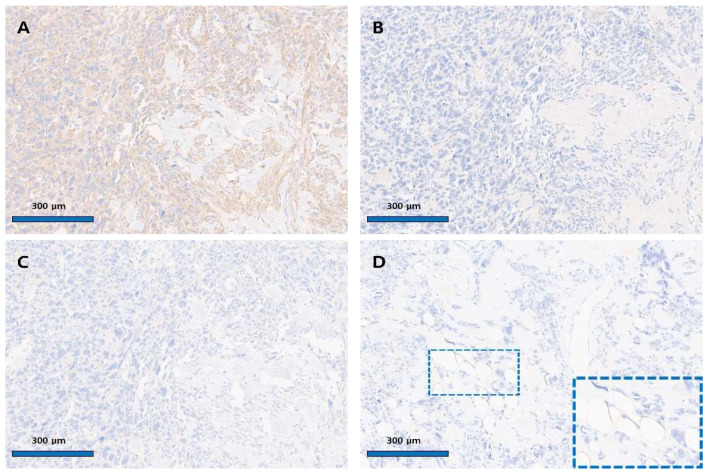
Immunohistochemical finding of the uterine angiomyolipoma. (**A**) Diffuse strong cytoplasmic positivity for SMA in pleomorphic epithelioid cells (×100). (**B**) Negative immunoreactivity for HMB-45 (×100). (**C**) Negative immunoreactivity for Melan-A (×100). (**D**) Negative staining for S-100 in tumor cells (×100); blue inset shows S-100 positivity in adipose cells (×400). Immunohistochemical staining was performed using the Ventana Benchmark Ultra automated platform (Ventana Medical Systems, Tucson, AZ, USA) with ready-to-use, pre-diluted antibodies. Appropriate positive controls were included for each marker, and the results were interpreted by comparison with internal or external control tissues. The following primary antibodies were used: SMA (clone 1A4, Cell Marque, Rocklin, CA, USA; Cat. No. 05268303001; internal control: smooth muscle cells), HMB-45 (clone HMB-45, Ventana, Tucson, AZ, USA; Cat. No. 790-4366; external control: melanoma), Melan-A (clone A103, Ventana, Tucson, AZ, USA; Cat. No. 790-2990; external control: melanoma), S-100 (clone 4C4.9, Ventana, Tucson, AZ, USA; Cat. No. 790-2914; external control: neurogenic tumor), Ki-67 (clone 30-9, Ventana, Tucson, AZ, USA; Cat. No. 790-4286; internal control: nuclei of tumor cells), and CD31 (clone JC70, Cell Marque, Rocklin, CA, USA; Cat. No. 05463475001; internal control: endothelial cells). Positive and negative staining patterns were determined by comparison with the corresponding control tissues (Figure 4). The pleomorphic epithelioid cells were diffusely and strongly positive for SMA. In contrast, they were negative for HMB-45, Melan-A, and S-100. S-100 positivity was observed in adipose cells, and CD31 positivity was seen in vascular endothelial cells. One mitosis was observed per 50 high-power field; no atypical mitoses were seen, and the Ki67 index was less than 5%. Tumor cell necrosis was not observed. Based on morphology and immune profile, the tumor was diagnosed as angiomyolipoma (PEComa subtype) with diffuse nuclear atypia. Nuclear atypia was interpreted based on nuclei exceeding three times the size of adjacent tumor nuclei, with pleomorphism, coarse chromatin, and prominent nucleoli. Although such atypia has been linked to aggressive behavior in renal AMLs, its significance in uterine AMLs is less established; in this case, the absence of necrosis, high mitotic activity, or an elevated proliferation index supported a non-aggressive interpretation. In the current case, the principal diagnostic challenge was to distinguish uterine angiomyolipoma (AML) from lipoleiomyoma. Unlike a typical leiomyoma that contains only focal or limited clusters of adipocytes, the present tumor demonstrated abundant mature adipose tissue diffusely admixed with medium-sized, hyalinized vascular structures throughout the lesion. The prominence of these thick-walled, tortuous vessels, together with the diffuse distribution of adipose tissue, was regarded as a decisive feature favoring a diagnosis of uterine AML rather than lipoleiomyoma. This characteristic vascular component, which represents one of the three histologic elements of the classic triphasic pattern of AML, was particularly instrumental in establishing the diagnosis. The final histopathology, given its rarity and atypical features, was presented and discussed at our institution’s multidisciplinary tumor board. Based on the final diagnosis of AML with atypia but otherwise benign features, the consensus was that no adjuvant therapy was warranted. The postoperative follow-up schedule consisted of regular physical examinations and transvaginal ultrasonography. These assessments were performed every three months for the first two years, and every six months for the subsequent three years. CT imaging was performed when clinical symptoms suggestive of recurrence (e.g., abdominal pain) were present, whereas routine annual CT scans were obtained for long-term surveillance during asymptomatic periods. The patient has been followed for 56 months post-surgery and, at the time of this report, shows no evidence of recurrence or metastasis. AML is a rare benign tumor. It primarily occurs in the kidney, liver, and lymph nodes, and is also known to rarely develop in the ovaries [1,2,3]. Among these, reports of AML arising in the uterus are extremely rare. According to a systematic analysis report from 2022, a total of 11 case reports were identified [4]. The current case differs from the previously published 11 cases in that it is accompanied by prominent diffuse nuclear atypia. Nuclear atypia is often observed in AML arising in the kidney and can pose challenges for differential diagnosis. Reporting nuclear atypia in uterine AML is believed to be the first such case. AML is a tumor composed of blood vessels, adipose tissue, and smooth cells, belonging to the PEC tumor family. Tumors belonging to the PEC tumor family are characterized by the expression of HMB-45, Melan-A, and SMA. HMB-45 and Melan-A are antigens highly expressed in melanocytes, and they play a crucial role in diagnosing melanocyte-derived tumors such as malignant melanoma or skin nevi [5,6,7]. Additionally, SMA is a useful antigen for diagnosing leiomyoma, a tumor of smooth muscle origin. PEC cells are characterized by expressing all three antigens—HMB-45, Melan-A, and SMA—making the diagnostic criteria relatively clear [5]. Additionally, SMA is a useful antigen for diagnosing leiomyoma, a tumor of smooth muscle origin. PEC cells are characterized by expressing all three antigens—HMB-45, Melan-A, and SMA—making the diagnostic criteria relatively clear. However, in uterine AML, HMB-45 expression is frequently absent, leading to differing opinions on this point. In 2021, Garg et al. reported that uterine AML can be divided into HMB-45-positive and HMB-45-negative groups. The HMB-45-positive group tends to be associated with tuberous sclerosis disease and has metastatic potential [8]. Conversely, they argued that the HMB-45-negative group does not show tuberous sclerosis and has a favorable prognosis, suggesting that HMB-45-negative uterine AML should be named angiolipoleiomyoma. According to the literature review [9,10,11,12,13,14], the majority of uterine AML cases show HMB-45 negative findings, with only two cases being HMB-45 positive and associated with tuberous sclerosis. The present case showed HMB-45 negative findings, and, notably, diffuse nuclear pleomorphism was prominently observed. In renal AML, such pleomorphic epithelioid features are known to be associated with a more aggressive clinical course [2]. However, in uterine AML, there are reports that the HMB-45-negative group exhibits benign behavior. Currently, this patient has been followed up for 56 months post-surgery, with no evidence of recurrence or metastasis observed. Although there is a limitation in that sufficient follow-up time has not yet been achieved, based on our case, there appears to be no association between diffuse nuclear pleomorphic features and an aggressive clinical course in HMB-45-negative uterine AML. Recent studies have proposed histologic algorithms, such as the Folpe criteria, to stratify the behavior of uterine PEComas based on size, mitotic activity, necrosis, and invasion [6]. Bennett and Schoolmeester later refined these models by incorporating immunophenotype, showing that HMB-45-negative, SMA-dominant tumors behave indolently, even when atypical [5,7]. In our case, the uterine tumor measures 6.7 × 8.2 cm with a mitotic rate of 1/50 HPF, absence of necrosis, no vascular invasion, and diffuse nuclear atypia. This profile fulfills the size criterion (>5 cm) and shows nuclear atypia but does not meet the thresholds for high mitotic activity (>1/50 HPF), necrosis, or vascular invasion. Under the Folpe/modified Folpe framework for gynecologic PEComas, such a lesion is most appropriately categorized as a PEComa of Uncertain Malignant Potential (PUMP), reflecting a limited but non-negligible risk of aggressive behavior and justifying careful long-term surveillance. Nuclear atypia alone should not be regarded as a predictor of aggressive behavior in HMB-45-negative uterine PEComas. Long-term follow-up and integration of molecular markers (TSC1/TSC2, TFE3) are recommended to improve prognostic accuracy [15,16]. We report a case of uterine AML without tuberous sclerosis in which HMB-45 was negative. The current case represents the first documented HMB-45-negative uterine angiomyolipoma with diffuse nuclear atypia, showing a low mitotic index, low Ki-67, and a benign 56-month outcome. It expands the morphologic spectrum of uterine PEComas, demonstrating that nuclear atypia alone does not imply malignancy in HMB-45-negative variants. Recognition of this variant is essential to prevent overdiagnosis and overtreatment, emphasizing the need for integrated radiologic–pathologic correlation and long-term surveillance. Analysis based on follow-up of a larger number of cases is considered necessary.

## Data Availability

The data presented in this article are available from the corresponding author upon request.

## Abbreviations

The following abbreviations are used in this manuscript:

AML	Angiomyolipoma
PEComa	Perivascular epithelioid cell tumor
SMA	Smooth Muscle Actin
HMB-45	Human Melanoma Black-45
